# Association between exposure to earthquake in early life and diabetes mellitus incidence in adulthood with the modification of lifestyles: Results from the Kailuan study

**DOI:** 10.3389/fped.2022.1046086

**Published:** 2022-11-08

**Authors:** Xinying Shui, Lei Zhao, Wenli Li, Yaning Jia, Ziquan Liu, Chen Li, Xueli Yang, Haoran Huang, Shouling Wu, Shuohua Chen, Jingli Gao, Xiaolan Li, Aitian Wang, Xiaobin Jin, Liqiong Guo, Shike Hou

**Affiliations:** ^1^ Institute of Disaster and Emergency Medicine, Tianjin University, Tianjin, Tianjin, China; ^2^ Wenzhou Safety (Emergency) Institute, Tianjin University, Wenzhou, China; ^3^ Tianjin Key Laboratory of Disaster Medicine Technology, Tianjin University, Tianjin, China; ^4^ Department of Occupational & Environmental Health, School of Public Health, Tianjin Medical University, Tianjin, China; ^5^ Basic Medical Science College, Harbin Medical University, Harbin, China; ^6^ Department of Cardiology, Kailuan General Hospital, Tangshan, China; ^7^ Department of Intensive Medicine, Kailuan General Hospital, Tangshan, China

**Keywords:** earthquake exposure, early life, diabetes mellitus, lifestyles, the Kailuan study

## Abstract

**Background:**

Exposure to disasters in early life may induce lifetime health risk, but investigation on earthquake exposure and DM in later life is still limited. The aim of the current study is to evaluate the association between exposure to the Tangshan Earthquake in early life and diabetes mellitus (DM) incidence in adulthood, and explore the modification of lifestyles on DM development.

**Methods:**

Participants who were free of DM at baseline from the Kailuan Study were included in this study. All participants were divided into fetal-exposed, infant-exposed, early childhood-exposed and nonexposed group. The effect of earthquake exposure on DM and modification of lifestyles were examined by multivariable-adjusted Cox proportional hazard model.

**Results:**

The exposed group had a higher risk of DM than nonexposed group, especially in infant-exposed and early childhood-exposed group, with hazard ratio (HR) of 1.62 [95% confidence intervals (CI), 1.21–2.17] and 1.46 (95% CI, 1.06–1.99), respectively. After stratifying by lifestyles, a significant modification was observed in alcohol consumption.

**Conclusion:**

Exposing to earthquake in early life could increase DM incidence in later life, and alcohol consumption might modify the effect of earthquake exposure on DM development. More attention should be paid on the preventions of DM among adults who exposed to earthquake in their early life.

## Introduction

Diabetes mellitus (DM) is a common kind of metabolic disease globally with an estimation of 425 million afflicted individuals in 2017. The global prevalence of DM is increasing and expected to reach to 522 million by 2030 (1). As a metabolic disease, the origin of DM is complicated, including human genetic and lifestyle factors act to promote DM in adulthood (2). Previous studies have identified over 100 genetic loci variant associated with DM (3), and evidence suggested that the risk of DM incidence decreased by 57% among participants with healthy lifestyles, including no smoking, moderate alcohol intakes, physical activities and healthy diets, compared with participants with unhealthy lifestyles (2, 4).

Emerging evidence suggests that exposure to environmental factors may have an impact on human health, including short-term and long-term impact. For example, a study of 497 DM patients were found that the control of blood glucose had worsened in 3 months after the Great East Japan Earthquake, which indicated that the secretion of endogenous insulin induced by hyperactivity of sympathetic nerve in a relatively short term (5). Similarly, adverse experiences in childhood have also been identified as a critical risk factor of psychiatric disorders, such as depression and post-traumatic stress disorder (PTSD) in a short-term (6). It has been reported that a dose-response relationship exists between exposure to Ukraine famine (1932–1933) in prenatal development and the incidence of DM in adults, revealing the long-term impact of disaster events (7). Likewise, the “Developmental Origins of Health and Disease” (DOHaD) hypothesis provide a potential link through biological reactions to early life exposure that are posited to predispose individuals to metabolic diseases (8). However, the pathways from exposure factors affecting early life to outcomes in adult life are far from clear due to the lack of longitudinal cohort studies.

Earthquake is a kind of severe natural catastrophic event that can cause adverse physiological and mental responses by fractures, crush injuries, and the severe damages of their properties and loss of their relatives (9). Studies on earthquake experience confirmed that earthquake trauma could increase the risk of DM. A study of Kumamoto Earthquake revealed that the glycemic deterioration after earthquake could be explained by increased production of cortisol and/or catecholamine (10). In addition, along with the rapid development of China, the lifestyles of Chinese people have changed significantly. A previous study has pointed out that less ideal cardiovascular health metrics might increase the risk of CVD in the population, who have been exposed to famine during the fetal period (11). However, little longitudinal cohort studies have examined the DM risk in adults who have exposed to earthquake during early life, and the interaction of earthquake exposure and healthy lifestyles on DM development are also few investigated.

Thus, we use the Tangshan Earthquake as a natural exposure, which happened in Hebei province, China, with an epicenter at Tangshan city on July 28 in 1976, a magnitude level of 7.8 on the Richter scale. The survey data were collected from the Kailuan Study, a community-based cohort in Tangshan. We aimed to examine the association between exposure to the Tangshan Earthquake during early life and the incidence of DM, and explore the potential modification of lifestyles on DM development.

## Materials and methods

### Study population

The Kailuan study is an ongoing prospective cohort (trial registration number: ChiCTR-TNC-11001489) study in Tangshan, China (12, 13). In 2006 to 2007, a total of 169,548 participants (≥18 years, including retired individuals) from Kailuan Group received questionnaires and the first health examination at Kailuan General Hospital and 10 affiliated hospitals. Following surveys were provided every 2 years since 2006 (the median of follow-up years was 6.07 years).

**Figure 1:**
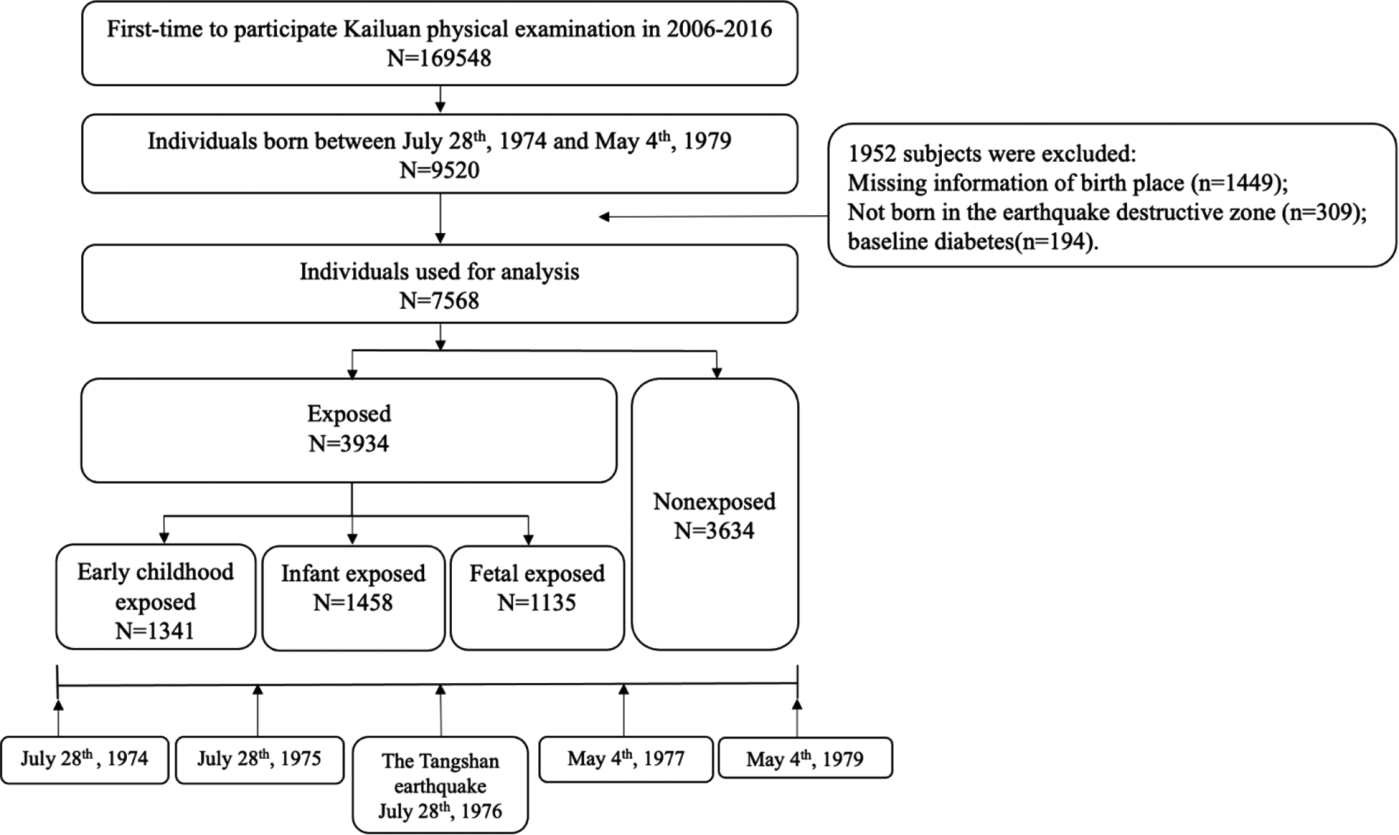
Flowchart of samples collection.

In this study, we included participants who participated in at least one survey from 2006 to 2017 and were born in the destructive zone of the Tangshan earthquake between July 28th, 1974 and May 4th, 1979. And the birth location of participants was collected based on the information of identification card. Inclusion criteria for birth dates was based on the Tangshan earthquake date (July 28th, 1976) and gestation period of 280 days. According to the date of the Tangshan earthquake and birth date of each participant, participants born between May 4th, 1977 and May 4th, 1979 were not exposed to the earthquake, who were defined as nonexposed group, and participants born between July 28th, 1974 and May 4th, 1977 were exposed to earthquake in gestation or early life, who were defined as exposed group. Meanwhile, exposed group were further divided into fetal-exposed group with participants born between July 28th, 1976 and May 4th, 1977, infant-exposed group with participants born between July 28th, 1975 and July 28th, 1976, and early childhood-exposed group with participants born between July 28th, 1974 and July 28th, 1975. The criteria for excluded were as follow: (1) participants who were not born in the destructive zone of the Tangshan earthquake; (2) participants without location of birth; (3) participants with DM at the baseline survey. Ultimately, a total of 7,568 eligible participants were included in this study. More details of the included and excluded individuals are shown in Figure 1. The study followed the Declaration of Helsinki and was approved by the Ethics Committee of the Kailuan Medical Group. All participants gave their written informed consent.

### Earthquake exposure

The Tangshan earthquake was a natural disaster resulting from a magnitude 7.6 earthquake that hit the region around Tangshan, Hebei, China on July 28th, 1976. It is a kind of severe natural catastrophic event that can cause adverse physiological and mental responses. In this study, earthquake exposure was defined based on the date of the Tangshan earthquake and birth date of each participant. Earthquake severity was measured by seismic intensity according to the New Chinese Seismic Intensity Scale in 1957, which varied from I (not felt) to XI (disastrous), and the destructive zone was comprised of area where the intensity was between destructive and disastrous (V–XI) (9, 14). All participants who had similar characteristics of socio-economic status and living habits were selected from the destructive zones of the Tangshan earthquake.

### Definitions of lifestyles status at baseline

The demographic characteristics (including gender, age, date of birth, education level and earthquake bereavement), lifestyles (including smoking status, alcohol intake, physical activity, sedentary behaviors and salt intake) and medical history of participants were collected face-to-face by trained staff using a standard questionnaire, which had been described in previous research (15), the earthquake bereavement was also collected through a structured questionnaire, with the following question, “Did you lose any relatives in the earthquake?”. Healthy lifestyle score was assigned for each component of the five predefined lifestyles, with 0, 1, and 2 representing poor, intermediate, and ideal levels, respectively. The combined lifestyle score was the sum of the 5 lifestyle factors with a range from 0 to 10. Participants with a score of 0 was considered the worst possible scenario and those with 10 was considered the optimal scenario, and total score were divided into three quartiles, poor, intermediate and ideal (15). More details were presented in Supplementary Table S1.

Current smokers were defined as individuals who have smoked at least one cigarette per day over the last 6 months. Past smokers were defined as individuals who have quit smoking before or during the baseline survey. Meanwhile, current drinkers were defined as individuals who have drunk at least once a month over the past year, and past drinkers were defined as individuals who have quit drinking before or during the baseline survey. The sedentary behavior was defined as poor, intermediate or ideal with a relative sedentary time of ≥8, 4–7 and <4 h/day, respectively. For individuals who had physical activity 1–2 times per week and lasted for more than 20 min per time were defined as intermediate physical activity. The diet status of individuals was defined as poor, intermediate and ideal based on salt intake of ≥10, 6–9 and <6 g/day, respectively (15).

### Covariates at baseline

DM was defined as either a self-reported physician diagnosis, or taking antidiabetic medication, or fasting blood glucose (FBG) ≥7.0 mmol/L in physical examination (16). Self-report of a physician diagnosis and an antidiabetic medication were collected by questionnaires provided by the survey among Kailuan General Hospital and 10 affiliated hospitals participants. Fasting blood glucose was measured using the hexokinase/glucose-6-phosphate dehydrogenase method (Mind Bioengineering Co Ltd., Shanghai, China) (17). Blood pressure was measured in the morning using physical examination, coffee, tea or physical exercise and other behaviors may affected the blood pressure were prohibited within 30 min before measurement. The measurement of blood pressure was repeated for 3 times, with 1–2 min interval for each measurement, and the mean value of blood pressure was taken. The height and weight of participants were measured by trained nurses, and body mass index (BMI) was calculated as weight (kg)/height^2^ (m^2^). Biochemical evaluation used the same fasting blood sample taken in the morning. Concentrations of low-density lipoprotein cholesterol (LDL-C), high-density lipoprotein cholesterol (HDL-C) and triglycerides (TG) were measured at Kailuan General Hospital and 10 affiliated hospitals using an autoanalyzer (Hitachi 747; Hitachi, Tokyo, Japan).

### Statistical analyses

For baseline characteristics of the study participants, mean ± standard deviation (*χ* ± *s*) or median values with interquartile range (IQR) were used for continuous variables. Number and percentage (%) were used to describe categorical variables. Kolmogorov–Smirnov tests were performed for checking data normality. Pearson *χ*^2^ test for categorical variables and Student *t* test or Mann-Whitney *U* test for continuous variables were used to compare the characteristics of the participants across baseline groups. The follow-up period was defined from the baseline survey to the onset of DM or the last visit on December 31th, 2017.

The comparison of the DM incidence with earthquake exposure was calculated by multivariate cox regression model, and hazard ratios (HR) with 95% CI were also calculated, with nonexposed individuals as the reference category. Except for all exposure and outcome variables, the missing covariates were imputed by multiple imputation methods (15). Model 1 adjusted for gender (male or female). Model 2 additionally adjusted for body mass index (≥24 or <24 kg/m^2^), high education level (below high school or high school or above), income level (more than 5,000¥), earthquake bereavement (no, yes) and healthy lifestyle score (poor, intermediate, ideal). Model 3 included Model 2 and additionally adjusted for systolic blood pressure (SBP) (continuous variables), diastolic blood pressure (DBP) (continuous variables), TG (continuous variables), HDL (continuous variables), and LDL (continuous variables).

To demonstrate the possible modification of lifestyles in the development of DM, we generated interaction terms using the cross products of earthquake exposure with healthy lifestyle score, smoking status, alcohol consumption, physical exercise, sedentary time, and salt intake into multivariate cox regression model, respectively. The interactions between the lifestyles and DM development were examined by likelihood ratio testing. The *P* for interaction was calculated based on the number of exposure groups (nonexposed and exposed) and the number of subgroups for each modificator in subgroup analysis. The sensitivity analysis was performed to examine the effect of earthquake bereavement. We analyzed the 2014 and 2016 bereavement questionnaires (*n* = 6,373), and further compared the risk of DM with stratification of non-bereaved and bereaved earthquake survivors, with the nonexposed group as the reference. Furthermore, we compared the risk of incident DM with stratification of earthquake exposure (fetal-exposed, infant-exposed, and early childhood-exposed group) with the nonexposed group as the reference category. As the incidence of DM and other factors is not linearly increase with age, and the age difference between born during the earthquake and post-earthquake may introduce substantial bias in analysis. Many previous studies have employed the age-balance method to minimize the bias between earthquake (exposed group) and post-earthquake births (nonexposed group) (18–20), which was conducted by combining the pre-earthquake births and post-earthquake births as a new control group.

All analyses were conducted using SAS (Version 9.4; SAS Institute, Cary, NC), and a two-tailed *P* < 0.05 was considered as statistically significant.

## Results

Among 169,548 individuals who participated Kailuan physical examinations between 2006 and 2017, 9,520 participants were born between July 28th, 1974 and May 4th, 1979. After excluding participants who had missing information of birth places (*n* = 1,449), were not born in earthquake destructive zone (*n* = 309) and had DM at the baseline (*n* = 194), 7,568 participants were included in this study (Figure 1). Among them, 6,111 (81.37%) were males, and the mean age was 33.5 ± 4.0 years. As shown in Table 1, compared with participants in the nonexposed group, participants in the exposed group were significantly older and had higher average SBP and DBP (*P* < 0.05). The distributions of high education level and earthquake bereavement were significantly different in two groups (*P* < 0.05) In addition, the baseline characteristics were almost comparable between participants included and those excluded due to missing birth places information, except that participants without birth places information had a lower proportion of males (Supplementary Table S2).

**Table 1:** Baseline characteristics by different earthquake exposure groups.

	Earthquake nonexposed	Earthquake exposed	*F/χ* ^2^	*P*
Date of birth	May 4th, 1977-May 4th, 1979	July 28th, 1974-May 4th, 1977		
Participates, *n* (%)	3,634 (48.02)	3,934 (51.98)		
Age at survey (*χ* ± *s*, year)*	32.3 ± 3.9	34.7 ± 4.0	−27.17	<0.01
Male, *n* (%)	2,957 (81.37)	3,154 (80.17)	1.67	0.2
Smoking (%)	1,641 (45.16)	1,707 (43.39)	2.32	0.13
Drinking (%)	1,673 (46.04)	1,787 (45.42)	0.26	0.61
Salt preference (%)	630 (17.34)	678 (17.23)	0.05	0.98
Sedentary (%)	302 (8.31)	347 (8.82)	1.23	0.54
Regular physical exercise, *n* (%)	334 (9.19)	329 (8.36)	2.72	0.26
High education level, *n* (%)	1,914 (52.67)	1,895 (48.17)	15.12	<0.01
High income, *n* (%)	136 (3.74)	157 (3.99)	0.89	0.64
Earthquake bereavement, *n* (%)	147 (6.43)	251 (9.16)	12.36	<0.01
Body mass index (*χ* ± *s*, kg/m^2^)*	24.6 ± 3.8	24.7 ± 3.6	−0.92	0.36
Systolic blood pressure (*χ* ± s, mmHg)*	121.1 ± 15.4	122.1 ± 15.5	−3.01	<0.01
Diastolic blood pressure (*χ* ± s, mmHg)*	79.1 ± 10.0	80.1 ± 10.2	−4.36	<0.01
Total cholesterol (*χ* ± *s*, mmol/L)*	4.7 ± 1.1	4.7 ± 1.1	−1.33	0.18
Triglycerides (IQR, mmol/L)^†^	1.2 (0.8,1.9)	1.2 (0.8,1.9)	−0.41	0.68
Low-density lipoprotein (*χ* ± *s*, mmol/L)*	2.5 ± 0.8	2.5 ± 0.8	−0.91	0.36
High-density lipoprotein (*χ* ± *s*, mmol/L)*	1.5 ± 0.4	1.5 ± 0.4	−1.57	0.11

*Data are presented as mean ± SD or percentage.

^†^ IQR denotes interquartile range.

As shown in Table 2, 365 (4.82%) DM cases were identified during a median 5.8 years of follow-up, and the incidence density of DM was 6.59/1,000 person-years and 9.91/1,000 person-years for the nonexposed and exposed groups, respectively. The proportional hazard assumption for the Cox model was tested and found valid (*P* = 0.32). Compared with the nonexposed participants, the exposed participants had increased risks of DM in adulthood with HR of 1.47 (95% CI, 1.19–1.82) after multivariable adjustment in Model 3. Furthermore, in the age-balance analysis, the group combined of pre-earthquake and post-earthquake births was set as reference group, the increased risk of DM in exposed group remained statistically significant [HR 1.24, 95% CI, (1.05–1.48)] (Supplementary Table S3).

**Table 2:** Association between earthquake exposure and risk of DM in adulthood.

Model	Nonexposed	Exposed
Case subjects/total number	140/3,634	225/3,934
Incidence	6.59	9.91
Crude Model	1.000	1.50 (1.22–1.85)
Adjusted Model 1	1.000	1.53 (1.24–1.89)
Adjusted Model 2	1.000	1.46 (1.15–1.85)
Adjusted Model 3	1.000	1.47 (1.14–1.84)

Adjusted model 1 was adjusted for gender (male or female). Adjusted model 2 included adjusted model 1 plus body mass index (≥24 or <24 kg/m^2^), high education level (less than high school or high school or above), income level (more than 5,000¥), earthquake bereavement (no, yes), healthy lifestyle (poor, intermediate, ideal). Adjusted model 3 included adjusted model 2 plus systolic blood pressure (continuous variables), diastolic blood pressure (continuous variables), triglycerides (continuous variables), high-density lipoprotein (continuous variables), low-density lipoprotein (continuous variables).

The interactions of lifestyles and earthquake exposure in early life on incidence of DM were presented in Table 3. According to the healthy lifestyle score, all participants were further stratified to poor, intermediate and ideal. After the stratified analysis, the results showed that no interaction effect was observed in healthy lifestyle score (*P*-interaction = 0.23). However, a positive modification effect was found in an individual lifestyle factor, alcohol consumption (*P*-interaction < 0.05), and the HR of drinkers and non-drinkers were 1.82 (95% CI, 1.31–2.52) and 1.14 (95% CI, 0.80–1.62), respectively. Sensitivity analysis showed that the incidence density of DM was 10.44/1,000 person-years and 11.35/1,000 person-year for the non-bereaved and bereaved survivors, respectively (Table 4). Compared with nonexposed participants, the non-bereaved and bereaved survivors had increased risks of DM in adulthood with HR of 1.52 (95% CI, 1.21–1.90) and 1.71 (95% CI, 1.08–2.68) after multivariable adjustment in Model 3.

**Table 3:** Multivariable-adjusted HRs (95% CIs) for the association between earthquake exposure in early life and DM according to subgroup analysis among participants.

Model	Case subjects/*n*		HR (95% CI)	*P*-interaction
Healthy lifestyle score		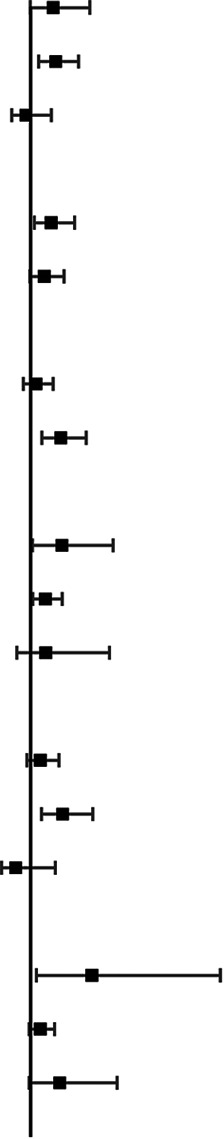		0.23
Poor	93/1,598		1.61 (0.99–2.62)	
Intermediate	209/4,923		1.68 (1.22–2.31)	
Ideal	63/1,047		0.87 (0.49–1.57)	
Smoking status				0.46
Non-smokers	187/4,220		1.56 (1.10–2.20)	
Smokers	178/3,348		1.37 (0.98–1.91)	
Alcohol consumption				<0.05
Non-drinkers	154/4,108		1.14 (0.80–1.62)	
Drinkers	211/3,460		1.82 (1.31–2.52)	
Physical exercise				0.81
No	75/1,544		1.85 (1.05–3.25)	
Occasionally	256/5,361		1.40 (1.06–1.86)	
Regular	34/663		1.41 (0.63–3.15)	
Sedentary time, h/day				0.13
<4	177/3,235		1.26 (0.90–1.78)	
4–8	166/3,684		1.87 (1.30–2.70)	
≥8	22/649		0.59 (0.21–1.68)	
Diet, based on daily salt intake (g/day)				0.06
<6	38/893		2.67 (1.16–6.18)	
6–9	268/5,367		1.26 (0.96–1.66)	
≥9	59/1,308		1.79 (0.96–3.36)	

**Table 4:** Association between earthquake bereavement and risk of DM in adulthood.

Model	Nonexposed	Not bereaved by earthquake	Bereaved by earthquake	*P* for trend
Case subjects/total number	140/3,634	165/2,488	22/251	<0.001
Incidence	6.59	10.44	11.35	
Crude Model	1.000	1.63 (1.30–2.04)	1.67 (1.06–2.62)	
Adjusted Model 1	1.000	1.61 (1.29–2.02)	1.74 (1.11–2.73)	
Adjusted Model 2	1.000	1.55 (1.23–1.94)	1.65 (1.05–2.59)	
Adjusted Model 3	1.000	1.51 (1.20–1.89)	1.68 (1.07–2.63)	

Adjusted model 1 was adjusted for gender (male or female). Adjusted model 2 included adjusted model 1 plus body mass index (≥24 or <24 kg/m^2^), high education level (less than high school or high school or above), income level (more than 5,000¥), healthy lifestyle (poor, intermediate, ideal). Adjusted model 3 included adjusted model 2 plus systolic blood pressure (continuous variables), diastolic blood pressure (continuous variables), triglycerides (continuous variables), high-density lipoprotein (continuous variables), low-density lipoprotein (continuous variables).

To explore the sensitive window during early life, the exposed group was further stratified to fetal-exposed, infant-exposed, and early childhood-exposed group based on the birth date and earthquake date. The incidence density of DM was 8.76/1,000 person-years, 11.29/1,000 person-years and 9.37 person-years for the fetal, infant and early childhood, respectively. Compared with nonexposed participants, infant and early childhood had increased risks of DM in adulthood with HR of 1.62 (95% CI, 1.21–2.17) and 1.46 (95% CI, 1.06–1.99) after multivariable adjustment in Model 3 (Table 5).

**Table 5:** Association between stratification of earthquake exposure and risk of DM in adulthood.

Model	Nonexposed	Fetal	Infant	Early childhood
Case subjects/total number	140/3,634	57/1,135	95/1,458	73/1,341
Incidence	6.59	8.76	11.29	9.37
Crude Model	1.000	1.31 (0.96–1.78)	1.74 (1.34–2.25)	1.42 (1.07–1.88)
Adjusted Model 1	1.000	1.30 (0.95–1.76)	1.77 (1.37–2.30)	1.48 (1.12–1.97)
Adjusted Model 2	1.000	1.24 (0.87–1.76)	1.64 (1.23–2.20)	1.46 (1.07–2.00)
Adjusted Model 3	1.000	1.21 (0.85–1.72)	1.63 (1.22–2.18)	1.45 (1.06–1.98)

Adjusted model 1 was adjusted for gender (male or female). Adjusted model 2 included adjusted model 1 plus body mass index (≥24 or <24 kg/m^2^), high education level (less than high school or high school or above), income level (more than 5,000¥), earthquake bereavement (no, yes), healthy lifestyle (poor, intermediate, ideal). Adjusted model 3 included adjusted model 2 plus systolic blood pressure (continuous variables), diastolic blood pressure (continuous variables), triglycerides (continuous variables), high-density lipoprotein (continuous variables), low-density lipoprotein (continuous variables).

## Discussion

To the best of our knowledge, this is the first study investigated associations between earthquake exposure in early life and risk of DM in adulthood in Asian populations. Moreover, it is also the first report about the effect of earthquake bereavement and the interaction of lifestyles and earthquake on the incidence of DM in adulthood. Our findings indicated that participants in the Tangshan earthquake exposed group were inclined to develop DM in adulthood. No interaction effect was observed between earthquake exposure and healthy lifestyle score, but an individual lifestyle factor, alcohol consumption was found that had positive modification effect on DM development. Meanwhile, compared with nonexposed participants, the bereaved survivors had increased risks of DM in adulthood, and the infant and early childhood period were specific susceptible earthquake exposure windows of DM development.

Supported by the DOHaD hypothesis, previous studies have linked exposure to disaster events in early life to metabolic disorders in adulthood. Lumey et al. showed that fetuses and children exposed to the Ukraine famine of 1932–33 was associated with an increased risk of DM in adulthood, with an odds ratio (OR) of 1.47 (95% CI 1.37–1.58) and 1.26 (95% CI 1.14–1.39) in extreme famine regions and severe famine regions (7). Dongfeng-Tongji cohort adequately demonstrated that the exposure to the Chinese famine (1959–1961) was associated with DM in adulthood during the 5 years follow-up period (21). As for earthquake, many studies reported the short-term impact of the Great East Japan Earthquake in Japan on DM development. It has been proved that the glycated hemoglobin (HbA1c) of affected individuals has altered and that glycemic control has worsened in a few months after the Great East Japan earthquake (5, 10, 22). In current study, the long-term impact of earthquake exposure on DM incidence in adulthood was investigated. Our results indicated that the early life stress indeed have impact on DM development, and the incidence of DM in adulthood among individuals who exposed to earthquake in early life was 1.47 times higher than nonexposed individuals, which was consistent with studies above. Moreover, it is worth mentioning that age difference is highly related to the incidence of DM, which may introduce substantial bias in analysis (18). Thus, the age-balance method was emplyeed to overcome this issue. After the age-balance analysis performed, the results still indicated a significant association between exposed group and combined group of pre-earthquake and post-earthquake. However, the development of DM may be affected by many factors, such as lifestyles and psychological stress, which need to be adjusted to obtain more accurate results.

Previous epidemiological and experimental studies have found that lifestyles may have impact on the risk of DM. Based on the China Cardiometabolic Disease and Cancer Cohort, Lu et al. found a significant interaction between ideal cardiovascular health metrics (ICVHMs) and famine exposure in early life on the risk of DM (18). Kautzky-Willer et al. reported that health behavior and physical activity were closely associated with risk of DM (23). In present study, the participants were divided into three quartiles (poor, intermediate and ideal) based on healthy lifestyle score, and we analyzed the interaction of healthy lifestyle score, individual lifestyle factor and earthquake exposure on DM incidence. However, no significant interaction was observed except alcohol consumption, and the results revealed that the increased risk of DM due to earthquake exposure might be modified by alcohol consumption in later life. Elgendy et al. conducted a study using data from the Evaluation of Diabetes Treatment study, an annual telephone survey in Quebec, Canada. The results implied the potential mechanisms between alcohol consumption and DM incidence, the chronic heavy alcohol consumption may induce alteration in glucose levels, insulin resistance, changes in lipid levels and interference in cell signaling (24–26), and individuals with DM who drink heavily were also more likely to have poor self-management behaviors, such as irregular diet, and seldom exercise, compared to those who drink less (27). Thus, alcohol consumption can increase the risk of DM through these metabolic, cell signaling and self-management changes. These findings have strongly supported our results. In addition, many previously epidemiologic studies showed the association between chronic diseases and bereavement stress among earthquake survivors. The prevalence of Post-Traumatic Stress Disorder after earthquakes ranged from 4.10% to 67.07% in adults and from 2.50% to 60.00% in children, and bereavement during the disasters is a predominant predictor (28). A cross-sectional survey showed that the bereaved survivors had a higher tendency to develop prolonged grief disorder (OR, 5.14; 95% CI, 2.72–9.74), compared with non-bereaved group (29). In present study, the bereaved survivors had increased risks of DM in adulthood after multivariable adjustment (*P* for trend <0.001), which indicated that more effective and sustainable mental health services were needed for bereaved survivors.

Amount studies have emphasized that the early life is a vulnerable period of development. Zhang et al. used the data from the Chronic Disease Survey and observed an increased risk of hyperglycemia in early childhood famine exposure cohort compared to the unexposed cohort with an OR of 1.46 (95% CI, 1.04–2.06) (30). Another study in Finland indicated that people evacuated overseas in early life during World War II had increment in the risk of cardiovascular disease and DM in late adulthood (31). To explore the sensitive window of early life, we further stratified the exposed group into fetal-exposed group, infant-exposed, and early childhood-exposed group, the results showed that infant and early childhood-exposed group appeared to have a significant increased risk of DM, compared with nonexposed individuals. However, the association between fetal-exposed group and risk of DM in adulthood was positive but not significant, which might be due to the inadequate sample size.

The mechanisms underlying the observed association between earthquake exposure in early life and DM in adulthood is difficult to determine and should be explored further. Evidence suggests that physiological stress response due to exposure to adversity in early life may be associated with concomitant activations of stress-related biological pathways (6, 32, 33). Although stress-related biological pathways (e.g., oxidative stress and inflammation) have been implicated in the development of diabetes and metabolic disorders (34, 35), the activation process of this system is not clear. It is likely to occur through dialogue with dysfunction of the hypothalamic-pituitary-adrenal (HPA) axis (36–40). Specifically, activation of the HPA axis by stress should generally lead to an increase in glucocorticoid sensitivity, enabling cortisol to inhibit and thus regulate inflammatory responses (39). Inflammation may promote the secretion of cortisol through compensatory mechanisms, and the basal secretion capacity of β cells and HDL-C may regulate the secretion of basal cortisol through negative feedback and lead to the onset and development of DM (36, 38). Nelson et al. reported that 6–12 months is specific time to developmental domains for HPA axis and this specific time is a critical period for the development of chronic metabolic diseases (6). Moreover, Shonkoff et al. found that the response when children exposed to adverse events, which called “toxic stress response”, may prolonged activation of the stress response systems that directly lead to dysregulation of the HPA axis and associated neuro-endocrine-immune as well as epigenetic effects (41). Another behavioral risk factor surveillance system data in U.S showed children exposed to high psychological stress have higher cortisol levels and greater risk of common diseases (33). These findings provided a convincible support to our results. Further studies are needed to follow up these participants continuously to investigate the relationship between earthquake exposure in early life and other metabolic diseases in later life.

The strengths of the current study include the large community-based cohort in northern China covered data on medical examinations, histories of disease, lifestyle factors, which could adjust potential confounding factors at individual level. In addition, the ages of eligible participants exposed to the Tangshan earthquake have been limited from fetal to 2 years old, which minimized the impact of aging on elevated risks of DM outcomes. However, there were several limitations to our study. First, in Kailuan study, the diagnosis of DM was based on a single measurement of FBG rather than oral glucose tolerance testing or the measurement of HbA1c, and therefore, the incidence of DM might have been underestimated. Second, our study did not distinguish between type 1 diabetes mellitus (T1DM) and type 2 diabetes mellitus (T2DM). According to the Clinical Guideline for Prevention and Treatment of T2DM in China, T2DM represents 95% of all cases of DM. Third, all participants were employees and retirees of the Kailuan Group, and 82.07% of participants were males, and we did not further stratify the analysis by gender. In addition, the territoriality of our northern occupational population is a restriction, the generalizability of the results is relatively limited. Finally, we acknowledged that the age differences would introduce bias in the development of DM. We have tried to minimize the impact of age differences as possible by using the age-balance method, which was commonly used in previous studies. However, the limitations of combining age group were still exist.

## Conclusion

In conclusion, the cohort study among Chinese populations suggests that exposure to earthquake in early life associated with DM in adulthood, and alcohol consumption and bereavement were all have impact on the incidence of DM. Furthermore, the significant association was observed in infant and early childhood-exposed group, which indicated that the infant and early childhood periods might be the sensitive windows of exposure period. These findings provide evidence on the adverse experiences in early life linked to DM in adults and emphasize the importance of enhancing health practice and sustainable mental health services among earthquake survivors to prevent early-stage chronic metabolic disorders.

## Data Availability

The original contributions presented in the study are included in the article/**Supplementary Material**, further inquiries can be directed to the corresponding author/s.
