# Supplementary material for: Association between exposure to earthquake in early life and diabetes mellitus incidence in adulthood with the modification of lifestyles: Results from the Kailuan study
Source: Front Pediatr. 2022 Nov 8;10:1046086. doi: 10.3389/fped.2022.1046086 (PMC9679373; doi:10.3389/fped.2022.1046086)
Supplement: Supplementary file 1 [file Datasheet1.docx]

Supplementary Material

**Table S1. Definition of Poor (0 Point), Intermediate (1 Point), and Ideal (2 Point) for Each Component of Healthy Lifestyle.**

| healthy Lifestyle Score Component | Poor (0 Point) | Intermediate (1 Point) | Ideal (2 Point) |
| --- | --- | --- | --- |
| Cigarette smoking | Current smoker | Past smoker* | Never |
| Alcohol drinking | Current drinker | Past drinker* | Never |
| Sedentary time, h/day | ≥8 | 4~7 | <4 |
| Physical activity (Moderate or vigorous exercise) | No physical activity | physical activity (≥20 minutes per time) 1-2 times per week, during leisure time | physical activity (≥20 minutes per time) 3 times per week, during leisure time |
| Diet, based on daily salt intake (g/d) | ≥10 | 6~9 | <6 |
| *Past smoker/drinker was defined when a participant who reported to smoke/drink previously but quit smoking/drinking during the survey. | | | |

**Table S2. Baseline characteristics of participants included and excluded due to missing information of birth places in the study.**

|  | Missing information of birth places | | | |
| --- | --- | --- | --- | --- |
|  | Yes | No | *F/χ2* | *P* |
| Participates, n (%) | 1417 (15.77) | 7568 (84.23) |  |  |
| Age at survey (‾χ ± s, year) | 34.5 ± 3.4 | 33.6±4.1 | 100.18 | <0.001 |
| Male, n (%) | 920 (64.93) | 6111 (80.75) | 174.64 | <0.0001 |
| Smoking (%) | 713 (50.32) | 3356 (44.34) | 16.94 | <0.0001 |
| Drinking (%) | 518 (36.56) | 3548 (46.88) | 50.95 | <0.0001 |
| Salt preference (%) | 187 (13.20) | 835 (11.03) | 12.17 | 0.0294 |
| Sedentary (%) | 269 (18.98) | 1310 (17.31) | 7.06 | 0.0294 |
| Regular physical exercise, n (%) | 154 (10.87) | 891 (11.77) | 12.49 | 0.0019 |
| High education level, n (%) | 809 (57.09) | 3782 (49.97) | 23.92 | <0.0001 |
| Earthquake bereavement, n (%) | 8 (5.06) | 398 (7.92) | 1.36 | 0.2435 |
| Body mass index (‾χ ± s, kg/m^2^) | 24.5 ± 3.8 | 24.6±3.7 | 2.89 | 0.0891 |
| Systolic blood pressure (‾χ ± s, mmHg） | 117.5 ±15.1 | 121.6±15.5 | 92.24 | <0.0001 |
| Diastolic blood pressure ‾χ ± s, mmHg） | 78.2 ±10.6 | 79.6±10.1 | 30.02 | <0.0001 |
| Total cholesterol (‾χ ± s, mmol/L) | 4.7 ± 1.0 | 4.7± 1.1 | 1.08 | 0.2977 |
| Triglycerides (IQR, mmol/L) ^†^ | 1.2 (0.8,2.0) | 1.2 (0.8,1.9) | 2.97 | 0.0846 |
| Low-density lipoprotein (‾χ ± s, mmol/L) | 2.5 ± 0.8 | 2.5±0.8 | 8.64 | 0.0033 |
| High-density lipoprotein (‾χ ± s, mmol/L) | 1.4 ± 0.4 | 1.5± 0.4 | 20.21 | <0.0001 |
| ^*^Data are presented as mean ± SD or percentage. | | | | |
| ^†^IQR denotes interquartile range. | | | | |

| Model | Pre-earthquake and post-earthquake births | Exposed |
| --- | --- | --- |
| Case/Number | 511/10641 | 231/3934 |
| Incidence | 4.80 | 5.63 |
| Crude Model | 1.00 (ref) | 1.20 (1.02-1.40) |
| Adjusted Model 1 | 1.00 (ref) | 1.21 (1.04-1.42) |
| Adjusted Model 2 | 1.00 (ref) | 1.23 (1.04-1.47) |
| Adjusted Model 3 | 1.00 (ref) | 1.24 (1.05-1.48) |
| Adjusted model 1 was adjusted for gender (male or female).  Adjusted model 2 included adjusted model 1 plus body mass index (≥24 kg/m^2^ or <24 kg/m^2^), high education level (less than high school or high school or above), income level (more than 5000¥), healthy lifestyle (poor, intermediate, ideal).  Adjusted model 3 included adjusted model 2 plus systolic blood pressure (continuous variables), diastolic blood pressure (continuous variables), triglycerides (continuous variables), high-density lipoprotein (continuous variables), low-density lipoprotein (continuous variables). | | |

**Table S3**. HRs (95% CIs) for incident DM according to earthquake exposure in early life among 14575 participants by age-balance analysis.
